# Quantifying the emergence of dengue inCE OF DENGUE IN HANOI, 1998-2009

**Published:** 2011-01-10

**Authors:** Hoang Quoc Cuong, Nguyen Tran Hien, Tran Nhu Duong, Tran Vu Phong, Nguyen Nhat Cam, Marcel Wolbers, Maciej F Boni, Peter Horby

**Affiliations:** 1 Oxford University Clinical Research Unit, Vietnam; 2 National Institute of Hygiene and Epidemiology, Hanoi, Vietnam; 3 Preventive Medicine Center of Hanoi, Hanoi, Vietnam; 4 MRC Centre for Genomics and Global Health, University of Oxford, Oxford, UK; 5 Centre for Tropical Medicine, Nuffield Department of Clinical Medicine, University of Oxford, Oxford, UK; 6 Pasteur Institute of Ho Chi Minh City, Vietnam

## Background

There was a large outbreak of dengue fever (DF)/ dengue hemorrhagic fever (DHF) with 16164 suspected cases in Hanoi in 2009 which was 5.2 fold higher than the same period of 2008. We aim to investigate if DF/DHF is really re-emerging in Hanoi since the last large outbreak in 1998 by observing the disease incidence and the average age of infection from 1998-2009 because the single bad year is not sufficient to conclude that dengue is re-emerging in Hanoi. Also, the standardized morbidity ratios (SMR) in 14 center districts of Hanoi in 2009 were calculated to identify the high-risk districts after correcting for demographic factors. We also look at the climate factors which can influence the dengue incidence in Hanoi.

## Methods

We analyzed 28479 cases reported from Preventive Medicine Center (PMC) of Hanoi and general population data from General Statistic Office (GSO). By using regression linear, we compared the average age infection by year. Poisson regression was used with year as a continuous variable to observe the incidence trend of DF/DHF in Hanoi. We used ArcGIS 9.3 to present SMR of DF/DHF between 14 districts Hanoi in 2009. We also used a Poisson regression to study the relationship between the climate factors such as temperature, rainfall, humidity and wind velocity and the incidence of Dengue in Hanoi. Linear regression model showed a slight increase in average of infection by year. Meanwhile, there was a significant upward trend of DF/DHF incidence from1998-2009 even we excluded two ‘bad years’ 1998 and 2009. Among fourteen center districts in Hanoi, three districts were identified as the high risk areas of DF/DHF which had SMR>3 in comparison with the average incidence of Hanoi. The maximum temperature, wind velocity and humidity were found to be correlated with the incidence of DF in Hanoi (p<0.05).

## Conclusions

The rising trend of DF/DHF incidence support the idea that dengue is emerging in Hanoi whereas the increase of age infection is not in favor of that idea. However, the age of infection have just increased slightly and did not show a clear trend so that we cannot exclude that dengue is re-emerging in Hanoi. The hot spots of DF/DHF in Hanoi were identified which located in the super-center districts with a very high density population.

